# Rebuilding research capacity in fragile states: the case of a Somali–Swedish global health initiative: Somali – Swedish Action Group for Health Research and Development

**DOI:** 10.1080/16549716.2017.1348693

**Published:** 2017-08-11

**Authors:** Abdirisak Ahmed Dalmar, Abdullahi Sheik Hussein, Said Ahmed Walhad, Abdirashid Omer Ibrahim, Abshir Ali Abdi, Mohamed Khalid Ali, Derie Ismail Ereg, Khadra Ali Egal, Abdulkadir Mohamed Shirwa, Mohamed Hussain Aden, Marian Warsame Yusuf, Yakoub Aden Abdi, Lennart Freij, Annika Johansson, Khalif Bile Mohamud, Yusuf Abdulkadir, Maria Emmelin, Jaran Eriksen, Kerstin Erlandsson, Lars L Gustafsson, Anneli Ivarsson, Marie Klingberg-Allvin, John Kinsman, Carina Källestål, Mats Målqvist, Fatumo Osman, Lars-Åke Persson, Klas-Göran Sahlén, Stig Wall

**Affiliations:** ^a^ Faculty of Medicine, Benadir University, Mogadishu, Somalia; ^b^ College of Health Sciences, Amoud University, Borama, Somaliland; ^c^ Faculty of Medicine, East Africa University, Bosasso, Somalia; ^d^ Medical College, University of Hargeisa, Hargeisa, Somaliland; ^e^ Medical College, Galkayo University, Galkayo, Somalia; ^f^ Medical College, Puntland University of Science and Technology, Galkayo, Somalia; ^g^ Somali–Swedish Researchers’ Association, Stockholm, Sweden; ^h^ Unit of Social Medicine and Global Health, Lund University, Lund, Sweden; ^i^ Division of Clinical Pharmacology, Karolinska Institutet, Stockholm, Sweden; ^j^ Dalarna University, Falun, Sweden; ^k^ Unit of Epidemiology and Global Health, Umeå University, Umeå, Sweden; ^l^ Department of Women’s and Children’s Health, Uppsala University, Uppsala, Sweden

**Keywords:** Somalia, fragile states, health systems, health research training, Diaspora

## Abstract

This paper presents an initiative to revive the previous Somali–Swedish Research Cooperation, which started in 1981 and was cut short by the civil war in Somalia. A programme focusing on research capacity building in the health sector is currently underway through the work of an alliance of three partner groups: six new Somali universities, five Swedish universities, and Somali diaspora professionals. Somali ownership is key to the sustainability of the programme, as is close collaboration with Somali health ministries. The programme aims to develop a model for working collaboratively across regions and cultural barriers within fragile states, with the goal of creating hope and energy. It is based on the conviction that health research has a key role in rebuilding national health services and trusted institutions.

## Background history and aims

In 1975, a special Swedish government agency was established for the promotion of research for development: the Swedish Agency for Research Cooperation with Developing Countries (SAREC). SAREC gradually established bilateral programmes of cooperation with low-income countries aiming at strengthening national research capacities. These programmes were mainly based on cooperation between national universities in these countries and in Sweden. Research students were assigned to projects identified and based in their home countries. They alternated fieldwork at home with periods for courses and for laboratory and data analyses in Sweden. At the time, this was termed ‘the sandwich model’ of research cooperation [1].

The research cooperation between Somalia and Sweden was agreed in 1981/1982 between the Somali Academy of Science and Art (SOMAC) and SAREC. Several faculties of the former Somali National University (SNU) became engaged in multi-sectoral research collaboration with more than 10 Swedish universities and research institutions. These partnerships in health, social, veterinary, and agricultural sciences lasted for 10 years until they were involuntarily disrupted by the civil war in the early 1990s.

The majority of the projects were within the health sector, addressing the Somali public-health challenges of that time. The collaboration targeted capacity building, and more than 20 Somali researchers were awarded Masters and/or doctoral degrees. Population-based research methods were applied in many projects, which motivated the inclusion of epidemiology courses [2]. After the outbreak of the civil war, several Somali researchers had to remain in Sweden to complete their theses. Some of them pursued a career in Sweden, while others were employed by international organisations. During the post–civil war era, they became engaged in the revival of medical education and allied health sciences institutions in Somalia/Somaliland.

To sustain the spirit of this partnership, the Somali–Swedish Researchers’ Association (SSRA) was established as a Swedish non-governmental organisation in 1993 with the aim of promoting research cooperation with Sweden. SSRA has supported several health interventions, working with local Somali civil society organisations and university academic institutions.

This paper outlines the reasons and motivations for relaunching a programme of Somali–Swedish research cooperation, which is now underway between six Somali and five Swedish universities. It aims, through close collaboration with health authorities, to rebuild health research capacity in Somalia. It may also serve to develop and test a model for working collaboratively with fragile states in general.

## Healing the Somali health system: needs, challenges, and opportunities

### The current situation

The protracted civil war and repeated conflicts since 1991 have devastated national institutions, producing more than one million refugees and an equal number of internally displaced people. The survival of the vulnerable population, predominantly mothers, children, and the elderly, was further jeopardised by the extended droughts and famines of 1991–1992 and 2011. Presently, in 2017, famine again threatens Somalia. In spite of international aid and protection provided by African Union Forces, many districts still lack sufficient access to vital health services.

As a result, the currently reported Somali health indicators are among the worst in the world, with infant and child mortality rates of 119 and 137 per 1,000 live births, respectively, and a maternal mortality ratio of 732 per 100,000 live births [3]. This is despite the Somali Joint Health and Nutrition Programme’s (JHNP) provision of an essential package of health services (EPHS) [4].

Inadequate public health–sector services have brought about a shift to the private sector, where both private health care and training institutions have grown, with the establishment of more than 20 health professional training programmes and about 14 medical colleges. Capacity building in these institutions constitutes a major imperative in view of the severe shortage of health workers. Still, the minimum number of 23 doctors, nurses, and midwives per 10,000 population, set by World Health Organization (WHO), are far from being achieved. The corresponding figures in Somalia are only 3–4 per 10,000 population [5].

These challenges were the focus of a National Health Conference, which was convened in 2013 in collaboration between the Benadir University, the Federal Ministry of Health, and the SSRA [6]. It was the first country-wide health conference for more than two decades, engaging close to 300 health professionals from all regions. Conference recommendations included the promotion of health research as an important tool to resolve challenges facing the health sector.

### The critical role of universities and health research in rebuilding fragile states

The rebuilding of fragile states requires that citizens regain possibilities to take care of their families and live a life free from war and other threats [7], and that they can rely on at least a minimum level of shared responsibilities for basic, social, and health services across ethnic, social, and economic groups [7,8].

In forming legitimate and trusted states, freedom from corruption, access to basic health and education facilities as well as security are fundamental [7–9]. Seven of the eight top-ranked fragile states globally are African, with Somalia as the most problematic case [8]. The ranking is based on 11 sub-scores covering grading of social, political, economic, and military pressures in a country [8]. Seven of these indicators are related to factors for which access to higher education and research at universities can make a difference: group grievance, brain drain, uneven economic development, poverty and economic decline, state legitimacy, public services, and human rights and the rule of law [8]. Few factors are reported to be as important as basic health with access to health services if a state is to gain trust and achieve legitimacy among its citizens [7,9,10].

Higher academic institutions in fragile states need to be part of the international movement for the free flow of ideas and experts between universities – in line with a declaration signed in Bologna in 1988 by 400 universities from across the globe [11]. It has been shown that university-based research has a major role in, for instance, the development of new medicines for diseases of priority in low-income countries [12]. Fragile and poor states will not be able to contribute to the prevention, diagnosis, and treatment of important health conditions and diseases unless they invest in research and higher education.

### Research for health: an essential function of the new Somali universities

Somalia started to develop institutions for higher education after independence in 1960, initially through links to Italian universities. Italian support was also important for the establishment, in 1970, of the SNU to meet the needs of nation building and socio-economic development. The SNU research capacity was enhanced by the partnerships established with Swedish universities in the early 1980s [1].

Health research has played an insignificant role in the private Somali universities and training institutions established in recent years. The main reasons for this include poor financial resources and the limited faculty and institutional capacity for research as well as the lack of a research culture.

Although a considerable amount of international aid is channelled to the health sector, concomitant support to universities and operational partnerships has remained marginal. Similarly, the Migration for Development in Africa (MIDA) Somalia project, which is engaged in mobilising Somali nationals in the diaspora to contribute to the development programmes, has also not focused on providing the needed academic expertise to these universities.

A positive recent development is that the Federal government has revived the SNU, launching several faculties, including a medical college, corroborating the public sector commitment to contribute to the health workforce development in the country. It is equally legitimate for the government to support academic institutions by attracting partnerships for the advancement of health research.

### The diaspora: an underutilised resource in health research and development

The Somali civil war of 1991 and subsequent internal conflicts prohibited health professionals from providing services in many regions, and led to the dilapidation of health facilities and the loss of large numbers of qualified health professionals. The conflicts also caused internal displacement of more than 1,000 health workers to safer urban centres, while another 600 doctors, nurses, and midwives, constituting about 20% of the workforce, migrated during the first five years of the civil war. This disruption was also accompanied by the closure of the only medical school in the country.

In 2000/2001, two medical schools – the Benadir University in Mogadishu and Amoud University in Borama – were founded by committed former faculty and alumni. In subsequent years, additional private universities have been established, often with the assistance of diaspora academics. Diaspora academics have also returned to the country and work as active faculty members in the medical schools and other health training institutions. Some young diaspora professionals have undertaken projects that are linked to their academic training in their countries of residence, for example in Sweden [13]. Moreover, the financial remittances from the Somali diaspora, estimated at US$1.43–2 billion yearly, are partially utilised for health services [14].

The Somali diaspora represents an important yet largely untapped resource for national academic training institutions [15]. Fostering partnerships with international organisations and universities, launching special programmes to finance guest positions for diaspora academicians (e.g. through MIDA), and promoting in-country operational links between Somali academic institutions and the service delivery system would all improve the scope and quality of the Somali health system.

### Communicating health information, policy, and research: the publishing divide

Limited access to research literature, as well as possibilities to communicate research results, is still a constraining factor for health development in low-income and fragile states. A Medline search of articles published during the past 25 years on health and nutrition subjects using the search words ‘Somalia’ and ‘Somaliland’ resulted in a total of 1,180 articles. When excluding those carried out in Somali diaspora communities, only 0.6% were co-authored by Somali scholars, illustrating the paucity of health research contributed by in-country Somali institutions and indigenous Somali researchers. Similarly, a Google scholar search for 2010–2016, also including grey literature, gave no more than 150 publications with ‘health’ and ‘Somalia’ or ‘Somali’ as part of the title, and few of these had Somali names as first authors. A national open-access health journal supported by diaspora academics would complement the envisaged research capacity building and provide the Somali researchers with a readily available platform to publish their quality research theses and reports, while also serving as a forum for sharing information on health development across the country.

## The present initiative for reviving Somali–Swedish cooperation in research for health

### Initial steps and joint planning process

Prior to the present initiative, the University College of Dalarna, the University of Hargeisa, and the University of Amoud in Somaliland initiated a cooperative programme in education and capacity building in the field of midwifery. Courses at master’s level, partly Internet based, were started in 2012 and yielded positive results and experiences. This programme motivated and inspired the present collaborative effort.

In 2014, the Unit of Epidemiology and Global Health (EpiGH) at the Umeå University and the SSRA decided to explore the possibilities for relaunching the previous research collaboration that was terminated at the outset of the Somali Civil War. Contacts were made with six different Somali universities and with Swedish universities that had participated in the previous Somali–Swedish research collaboration. A number of Somali diaspora health professionals were also contacted for the same purpose.

With some financial support from the Swedish International Development Cooperation Agency (Sida) via the Nordic Africa Institute, it was possible to organise a seminar in December 2014 [16] attended by 53 participants, including 25 Somalis. Six Somali universities (two in South Central Somalia, two in Puntland, and two in Somaliland) and six Swedish universities (Umeå, Uppsala, Lund, Dalarna, and Gothenburg as well as Karolinska Institutet) were represented. There were also participants from SSRA, Sida, and Forum Syd, an organisation that supports Swedish non-governmental organisations actively engaged in development projects.

The seminar outlined the challenges facing the health system, such as the shortage of health workforce and the poor health of the population. The importance of health-system strengthening, with a focus on maternal, neonatal, and child health, was underlined, and the role of academic institutions in advancing the link between health system policy and the use of health research to improve health services was emphasised.

The participants committed themselves to work for the promotion of national and international partnerships in support of Somali health development. They pledged to promote ‘health research as a key component of the national rebuilding process, to bridge the gap between knowledge and action in the country, and to contribute to developing the Somali primary health care system based on the principles of universal and equitable access to health and health care’. The statement *Healing the health system after civil unrest* was published [17] to raise awareness among and promote a response by the international community to address the needs facing the health sector in post-conflict situations. Ultimately, lessons learnt from the Somali situation may also guide health-sector development after civil unrest in other settings.

In November 2015, a follow-up workshop was held in Umeå with the aim of translating the aspirations set by the 2014 seminar into action, creating a platform for research capacity building [18]. It had 28 participants, representing three groups of partners: five Swedish and six Somali Universities, as well as the SSRA. The strong, active Somali participation is reflected by the fact that each of the participating Somali universities organised a pre-workshop seminar.

The participants deliberated on the need for enhancing analytical capacity, strengthening libraries’ information and communications technology (ICT), creating university budget lines to fund research, and establishing linkages with ministries of health. The workshop also identified the research priorities that will guide health research in the coming years. It emphasised the building of basic laboratory capacities and the creation of a Somali–Swedish health research training programme aimed at research that can be translated into action.

The 2014 and 2015 meetings have created lively contacts between the Somali universities across the politically diverse geographical zones, and offered the opportunity for internal consultations through Skype meetings for the first time ever. This has built trust across the Somali regions and has created opportunities for universities and university colleges to support the reconciliation processes within Somalia.

### Guiding principles and envisaged steps

We have committed ourselves to a working culture based on mutual respect and equal partnership. We have also recognised the importance of operationalising the work as a win–win collaboration, with clear, collective capacity-building benefits for both the Swedish and Somali partners. The benefits of the work will be maximised if it facilitates researchers to teach *and* teachers to conduct research.

The Somali partners – in particular those currently living and working in Somalia/Somaliland – should be the main driving force and play the main role in defining the direction of the training and research programme. The Ministry of Health and other key actors in the health sector should be involved in the different steps of the research process, from formulation of the research questions through to dissemination and action.

The overall mission of the programme is to strengthen research capacities and functions of Somali universities in response to priority health needs and to carry out research linked to policy and practice. The mission can be summarised as follows:

#### Developing basic skills for health research

An important goal is to ascertain that the Somali universities will have a cadre of faculty members with research skills in a broad sense. This includes the ability to define research problems, to review scientific literature, to design research using appropriate methodology, and to carry out research, as well as to compile and analyse data, which may have a bearing on policy and practice.

#### Promoting career development in research

The collaborative programme aims at building up a critical mass of researchers, which requires the establishment of possibilities for career development in research. It also includes the development of a research culture within the universities, which is conducive to scientifically sound research.

#### Strengthening infrastructures for research

Training for research goes hand in hand with doing research. Both activities depend on the availability of appropriate infrastructure in terms of physical assets such as offices and laboratories, computer facilities, as well as institutionalised administrative and regulatory functions.

#### Fostering communication and policy implications of research

A transparent research process with in-built communication between stakeholders and executers of research during all phases of the work will be an important aspect of the cooperative venture. Health research capacity building has important interdisciplinary and intersectoral aspects, which also motivates communications with administrative bodies other than those connected with the Ministry of Health. Scientific publishing has a critical role for sharing research results within the Somali health research community as well with policy and decision makers.

#### Establishing research collaboration at national as well as international level

The collaborative programme aims to act as a unifying force for the Somali university system. There is much to be gained from collaboration in all aspects of research development with joint planning and sharing of resources as well as in scientific contacts. The programme also aims to facilitate and promote contacts and collaboration over a wider span of actors at the international level.

#### Enabling the universities to harness the academic and health research potentials of the diaspora

The Somali health authorities and universities should recognise health research for action as a priority, identify strategies to engage the diaspora, and provide relevant facilitation incentives for their participation. Agreements with diaspora-hosting countries may be pursued to improve the skills and academic gaps perceived.

### What has been achieved?

The results of the collaboration between Dalarna University and their counterparts in Somaliland since 2014, also extended to a university in Puntland, have been encouraging. Altogether, 84 students have graduated, 59 with a master’s degree and 35 with a diploma in reproductive and perinatal health. A number of master’s theses have been published in international journals and have provided policy-relevant information [19–22]. Members of this partnership have also participated in the development of the present initiative.

To follow up the two meetings during 2014 and 2015, the Swedish partners met in Umeå in June 2016, while the Somali partners also met through Skype across their universities to discuss the scope of the first year of cooperation. This was followed by a joint Skype meeting when parties from both sides had an opportunity to compare notes and to agree on future activities.

It was decided to initiate the cooperative programme with a two-week research training course aimed at what was termed ‘training of trainers’ (TOT) at the Somali universities. It was agreed that each Somali university should recruit four to five trainees and also that a few trainees could be selected among ministry of health employees. The trainees would be expected to choose topics for a research project to be carried out over the following year. The selection process was carried out during the subsequent few months, together with teachers from the Swedish universities, following the development of a course curriculum and decision on time and venue. The course was to be organised in Hargeisa, Somaliland, where the local universities could offer logistic support and a safe surrounding.

The course took place 16–27 October 2016 [23]. It was attended by a total of 18 representatives from six Somali universities, three candidates from each, and one representative from each of the Federal Ministry of Health and from the Puntland and Somaliland ministries of health. Two additional candidates from Hargeisa University and the former Federal Minister for Health also joined the course.

The overall aim of the course was to teach the basics of epidemiological and qualitative research design and analysis, as well as the role of combining quantitative and qualitative approaches in public-health research. The course was designed to follow the research process from problem identification, planning, and data collection through analysis, interpretation, and documentation.

Supervisors were assigned to each trainee (one each from their respective Somali universities and one from the Swedish universities). The course offered trainees the opportunity to define their research questions and focus on issues that are of relevance for their specific situations. Following the initial two-week training, the trainees have 11 months to implement their research projects. During this period, researchers receive continuing support from their assigned Swedish and Somali mentors through online or face-to-face contacts, respectively. Two events of face-to-face contacts will be organised: one at the end of the first six months, where progress achieved and problems encountered will be discussed, and another at the end of the one-year programme, during which the trainees will present the findings of their research projects.

### Short- and long-term ambitions

Beyond the initial 12-month blended learning, a vision for a long-term collaboration has emerged. The vision is based on working within and influencing the health system and the Ministry of Health, thereby contributing to the national health system recovery through:

- The strongest trainees from the first batch going for further training;
- Training a second batch of trainees;
- Developing a PhD research programme based in Somalia;
- Developing a site for a Health and Demographic Surveillance System in each university region;

The potentials of an infrastructure for community-based research were illustrated in the 10-year research collaboration between EpiGH, UmU, and the Community Health Department in Mogadishu which started in 1982[24]. This work was led by Somali PhD students enrolled in the collaboration and gave them on-the-job training as well as material for their PhD theses.

We expect that the TOT course will result in a significant number of potential PhD candidates in the health sciences who can be enrolled in academic research training programmes with the collaborating Swedish partners and, later, be engaged in the development of Somali-hosted master’s and PhD programmes.

In the post-conflict phase of the Somali health-sector development paradigm, there is a need for a cadre of highly and well-trained researchers capable of helping to resolve the complex challenges facing the Somali health system. We focus on capacity-building programmes for younger scientists at PhD training and postdoctoral levels through twinning scholars within the same project, presuming there is senior supervision in place on both sides.

We envisage that in line with the above examples, a longitudinal field laboratory and demographic surveillance, with access to adequate biomedical laboratory resources, will serve to build a long-term research programme while at the same time be a training hub for the medical students as well as for master’s and PhD courses.

The six Somali universities have all committed to integrate research as one of the key pillars of their academic programmes, and they have agreed to allocate budgets for research support activities. The universities are also establishing their committees to deliberate on ethical issues related to research that involve human participants and/or personal data.

## The way forward

Our collaborative programme of health research and research training can be seen as preventive action, in its broadest sense, to counterbalance some aspects of the current global crisis, specifically those concerning refugees, poverty, and terrorism. This project represents a window of opportunity to show that well-designed and well-managed development aid can help to address the root causes of these problems.

The overall aim of the collaboration is to develop health research capacity as a means of improving health and healing the health system in Somalia and to

- Bridge the ‘know–do’ gap by seeking to link research training and research activities with policy and practice;

- Build a critical mass of researchers, including in the Ministry of Health, thereby creating a working culture in which research findings are seen as key for the improvement of population health and of the health system;

- Increase the number of university teachers with PhD degrees across the Somali region; secure and strengthen inter-university collaboration within Somalia and between Somali and Swedish universities;

- Facilitate the development of long-lasting collaborative partnerships between doctoral students and more established academics in Somalia and Sweden;

- Provide support for and motivation to disseminate research in scientific fora.

A basic principle of this collaboration is to take small, concrete steps at first – both as a means of getting to know each other and to demonstrate our capacities so that we can subsequently grow as we learn.

## Conclusions

When summarising the past 10-year project from the 1980s, stopped by external forces 25 years ago, we concluded that ‘some of the lessons learnt could be shared when the time comes to invest in another Somalia. It is then not just wishful thinking that health ought to be a major entry point for such a change’ [20]. This paper shares and revives basic principles for a sustainable collaborative venture between our three partners. We think that now is the time to take action.

Somalia is in a phase of mending the sores from the civil war while facing enormous political, socio-economic, and human-rights challenges. Health and nutrition services are being rebuilt but face a lack of human resources and infrastructure, as well as appropriate knowledge on the patterns of disease burden and much-needed cost-effective action programmes. The recent creation of universities and schools for training of professionals presents a potential resource and can make critical contributions if given appropriate support.

The contacts between Somali and Swedish universities during the last two years confirms the interest and commitments from both sides to engage in a programme of cooperation for health development and research based on Somali ownership and involvement of its diaspora.

The collaborating parties share the conviction that long-term cooperation over the next 5–10 years would be needed to secure a tangible outcome. Even if the universities on both sides have already manifested their ability to initiate a joint research training activity, a long-lasting effort would need support and funding from both national Somali sources, as well as from bilateral and international programmes for development cooperation.

Our initiative may have relevance for other fragile, post-conflict states [8], and our three-partner approach, involving domestic and external universities as well as diaspora academics, may prove to be a constructive model for international cooperation. There is an urgent need to find new ways of establishing hope and trust in the rebuilding of public institutions in fragile states. The academic communities at both domestic and international levels have a responsibility to engage in this process in the spirit of solidarity.

## Paper context

This paper summarises the background and motivation for reviving a pre–civil war research cooperation between Somali and Swedish universities. Despite protracted civil conflicts, Somalia is steadily recovering with motivated academic health institutions. To strengthen their ability to carry out research linked to policy and practice, a long-term programme is outlined also involving Somali diaspora academics. This may also serve as a model for cooperation with other fragile states.
